# Temporal Binding in Multisensory and Motor-Sensory Contexts: Toward a Unified Model

**DOI:** 10.3389/fnhum.2021.629437

**Published:** 2021-03-25

**Authors:** Kishore Kumar Jagini

**Affiliations:** Center for Cognitive and Brain Sciences, Indian Institute of Technology Gandhinagar, Gandhinagar, India

**Keywords:** temporal binding, multisensory, motor-sensory, causal inference, Bayesian models, precision

## Abstract

Our senses receive a manifold of sensory signals at any given moment in our daily lives. For a coherent and unified representation of information and precise motor control, our brain needs to temporally bind the signals emanating from a common causal event and segregate others. Traditionally, different mechanisms were proposed for the temporal binding phenomenon in multisensory and motor-sensory contexts. This paper reviews the literature on the temporal binding phenomenon in both multisensory and motor-sensory contexts and suggests future research directions for advancing the field. Moreover, by critically evaluating the recent literature, this paper suggests that common computational principles are responsible for the temporal binding in multisensory and motor-sensory contexts. These computational principles are grounded in the Bayesian framework of uncertainty reduction rooted in the Helmholtzian idea of unconscious causal inference.

## Introduction

We receive sensory information from the environment and the body through several distinct senses. For a coherent and unified representation of information, our brain needs to group the multisensory features emanating from an object or event (Calvert et al., 2004). For instance, imagine that you are applauding the musical performance of your friend by rhythmic hand clapping. The multiple sensory features (such as tactile, auditory, and visual) from hand-clapping are grouped and experienced as coming from a single causal event rather than separate events. Several challenges that the brain needs to overcome for grouping or often called “binding” the multisensory features of an event (Vroomen and Keetels, 2010; Vilares and Kording, 2011; Burwick, 2014; Spence and Frings, 2020). This paper focuses on two non-trivial and inter-related challenges that the brain must account for in binding the multisensory and motor-sensory features in the time domain.

The first challenge is causal determination. Our senses are bombarded with multiple sensory features that are either received passively or generated as a consequence of our motor actions. How does our brain deal with the ambiguity in matching sensory features that belong to one causal event and segregate others? Or how does our brain determine whether the sensory features are causal outcomes of our voluntary motor actions or not? The second challenge is with regard to the lack of precision in the temporal estimates of sensory features across the senses. This lack of precision in temporal estimates is assumed to be due to the noisy or uncertain sensory information and differential temporal resolution in the encoding of the temporal information across the senses (Kersten et al., 2004; Faisal et al., 2008; Vroomen and Keetels, 2010). How does our brain account for this sensory noise and differential precision in encoding the temporal information across the senses for coherent and robust perceptual binding of sensory signals coming from a common cause? Previous studies have proposed different mechanisms for the temporal binding phenomenon in multisensory and motor-sensory contexts (Haggard et al., 2002; Chen and Vroomen, 2013). This paper reviews the recent literature on the temporal binding phenomenon in multisensory and motor-sensory contexts. Moreover, this review suggests the existence of common computational principles grounded in the Bayesian framework for temporal binding in multisensory and motor-sensory contexts. The following section briefly describes various behavioral manifestations of the temporal binding and its constraints across the multisensory and motor-sensory contexts. After describing the basic temporal binding phenomenon, the author discusses the Bayesian inference models and the extent to which these models explain the temporal binding phenomenon in the multisensory and motor-sensory contexts.

## Temporal Binding and Temporal Binding Window

The term “temporal binding” refers to the subjective experience of mutual attraction between two or more events in the time domain. For example, in the audio-visual perception, the temporal aspect of a visual event, such as onset time, can be perceptually shifted and binds with a slightly asynchronous auditory event (Vroomen and Keetels, 2010; Chen and Vroomen, 2013). Similarly, in the motor-sensory contexts, the perceived onset times of self-generated motor action and its sensory outcome (e.g., visual or auditory event) are shown to be mutually attracted to each other (Haggard et al., 2002; Wolpe et al., 2013). The temporal binding phenomenon was also observed for other aspects of the time domain, such as frequency and duration. For instance, in the double-flash illusion, a single visual flash is perceived as multiple flashes when accompanied by multiple auditory beeps (Shams et al., 2000, 2005). With regard to the duration perception, studies have shown that visual events are perceived to be longer or shorter during the concurrent auditory event or motor action (Burr et al., 2009; Press et al., 2014; Anobile et al., 2019). Importantly, however, these temporal illusions are preserved over a time window known as “temporal binding window (TBW)” or “temporal integration window” (Diederich and Colonius, 2004; Wassenhove et al., 2007; Vroomen and Keetels, 2010). From the literature, it appears that there is a large variability in the extent of temporal binding windows across different combinations of paired multisensory stimuli, experimental paradigms, and stimulus (such as spatiotemporal, stimulus complexity) or cognitive factors (Andersen et al., 2004; Vroomen and Keetels, 2010; Stevenson and Wallace, 2013). Also, from the developmental perspective, studies have shown that the extent of multisensory temporal binding windows follows a U-shaped function with children and older age groups having larger binding windows compared to the young adults (Wallace et al., 2019). The increased temporal binding windows in children, older adults, and in certain neurodevelopmental disorders (e.g., autism) lead to the disruption of various cognitive abilities and reduced behavioral performance (Barutchu et al., 2010; Downing et al., 2015; Wallace et al., 2019).

## Bayesian Inference

In recent decades, studies from neuroscientific, behavioral, and computational approaches have indicated that the brain generates various mental events by “predictive-processing” of information (Rao and Ballard, 1999; Feldman and Friston, 2010; Clark, 2013; Hohwy, 2013; Hutchinson and Barrett, 2019). The core assumption of the “predictive-processing” framework is that the brain constantly runs an internal mental model of the world and uses it to predict the causes of the sensory effect. The internal model is assumed to be continuously updated based on the discrepancy between predicted and actual sensory input which is often referred to as prediction error (Raichle, 2015). The essential role of the brain is to minimize the prediction error for the best possible causal inference of sensory information. Although formal computational models of predictive processing frameworks have been developed recently, the core assumptions have roots in the Helmholtzian idea that the brain makes unconscious perceptual inference based on prior knowledge or prior learning (Von Helmholtz, 1867). One of the worrying problems for the perceptual inference is that there is no perfect one–one mapping between cause and sensory effect. Sensory information is corrupted with noise from the external world, noise in the nervous system, and variable precision of sensory encoding across the senses (Ernst and Bülthoff, 2004; Ernst, 2006). This variability in sensory information necessitates the brain to perform probabilistic (Bayesian) inference when computing predictions and prediction errors (Vilares and Kording, 2011). The main purpose of probabilistic processing is to update the internal models with precise prediction-error signals and ignore (or less prioritize) relatively less precise prediction-error signals. According to Bayesian probabilistic predictive processing models, perception arises from the precision-weighted probabilistic combination of prior belief or knowledge (or prior in Bayesian terms) of the world and the current sensory evidence (or likelihood in Bayesian terms). In other words, perception is determined by the trade-off between the precision of prior and likelihood.

In parallel lines, the characterization of cause-and-effect temporal relationships by Hume inspired numerous empirical studies to understand the predictive processing of the brain (Hume, 1739; Pearl, 1988, 2000; Hohwy, 2013). Hume has suggested that the inference of the relationship between cause and effect developed through statistical regularities in nature. He has proposed three fundamental cues that may support causal learning, such as temporal priority, contingency, and contiguity. Temporal priority refers to the idea that there must be an existence of cause before the sensory effect. This cause(s) and its effect(s) or causally related events are typically co-occurring together repeatedly and reliably (i.e., contingent) and co-occur close in space and time (i.e., contiguous). Numerous studies have experimentally manipulated the rules of causal learning to understand the causal learning and predictive processing of the brain and provided empirical evidence (Alais et al., 2010; Buehner, 2014).

Recent studies have suggested that the human temporal perception is consistent with Bayesian inference models across different time scales and temporal aspects (Shi et al., 2013; Rhodes, 2018). For example, a well-known perceptual phenomenon in the temporal dimension called “central-tendency effect” has been demonstrated to be quantitatively predicted by Bayesian inference models (Jazayeri and Shadlen, 2010).

### Bayesian Casual Inference in Multisensory Temporal Binding

Appropriate binding of multisensory features of an event and segregating others necessary for a coherent and unified perceptual representation lead to enhanced behavioral performance. For instance, previous researchers have demonstrated that the binding of multisensory information enhances the speed and accuracy of detection performance and increases the precision of sensory estimates that enhanced the discrimination performance (Ernst and Banks, 2002; Diederich and Colonius, 2004; Ernst and Bülthoff, 2004; Ernst, 2006).

The first challenge that our brain needs to account for in binding multisensory features of an event is solving the causal inference problem—determining whether sensory signals are coming from a common causal event or different events. That is, our perceptual system needs to infer the causal structure of the world from noisy sensory data for which we do not have direct access (Körding et al., 2007; Stein, 2012). Bayesian causal inference models explain how an observer might infer the causal structure for determining the probabilistic estimation of whether sensory signals are coming from a common causal event or different events (Körding et al., 2007; Wozny et al., 2010; Noppeney, 2020). The estimation or inference of causal structure is thought to be derived by the probabilistic averaging of the common cause prior (or prior knowledge that the signals are coming from a common source) and current sensory evidence according to the Bayesian models (Ernst, 2006, 2012; Körding et al., 2007). Therefore, the extent of binding or integration of multisensory signals depends on the strength of the inferred causal structure. For instance, forced fusion might happen only if an observer infers that the multiple signals are coming from a common cause with absolute certainty, or complete segregation of signals could happen if the observer infers signals are coming from separate sources. However, due to the inherent uncertainty of the sensory data and uncertainty in causal inference, the integration of multisensory signals can arbitrate between forced fusion and segregation (Körding et al., 2007; Shams and Beierholm, 2010; Ernst, 2012).

Previous research has indicated numerous cues that are suggested to act as common cause priors (e.g., spatial and temporal mapping or correlation between sensory signals) for solving the causal inference problem (Ernst and Bülthoff, 2004; Doehrmann and Naumer, 2008; Vroomen and Keetels, 2010; Buehner, 2014; Debats et al., 2017). For example, a typical multisensory event in the natural environment, a ball hitting a glass window, produces multiple sensory stimuli that are spatiotemporally proximal. These spatial and temporal regularities are utilized by our perceptual system to decide whether sensory cues are coming from the same or different causal events (Vroomen and Keetels, 2010; Chen and Vroomen, 2013). Moreover, the extant literature has indicated several higher-order cognitive factors such as semantic (Doehrmann and Naumer, 2008), metaphoric (Parise and Spence, 2009), or experimentally learned matching (Ernst, 2007) of paired multisensory cues involved in causal determination. This evidence also indicated that the causally related (e.g., congruent) multisensory features have a larger TBW than non-causally related (or unrelated) features. In other words, the larger TBW indicates that the casually related pairs of multisensory stimuli are more often perceived to occur together in time than the pairs of unrelated stimuli that have the same amount of asynchrony between them. Moreover, the strength of prior belief that the pair of events is causally related is shown to be positively correlated with the tendency to perceive events as co-occurring together in time (Faro et al., 2005).

The next question is how the brain optimally binds, in the time domain, the causally related multisensory features which are processed at different times due to the noise in the nervous system. According to the Bayesian causal inference models, the causally related multisensory features are temporally bound together by precision-weighted probabilistic cue combination (Vilares and Kording, 2011). In other words, the less precise sensory feature is perceptually shifted closer to the more precise sensory feature to maintain temporal coherence. For example, when the audio-visual cues of an object are presented asynchronously, the visual stimulus is perceived to occur temporally closer to the auditory stimulus, called “temporal ventriloquism” (Morein-Zamir et al., 2003). Since the precision of the temporal judgment of the visual cue is lower than the auditory cue, the Bayesian sensory cue combination predicts that the auditory temporal judgment is given more weight and shifts the visual stimulus perceptually closer and bound to the auditory stimulus (Alais and Weston, 2010; Chen and Vroomen, 2013). Similarly, Ley et al. (2009) showed that auditory and vibrotactile stimuli are perceptually bound according to the Bayesian cue combination. Other studies indicated that the semantic or learned correlations (congruent) between a pair of sensory cues induced greater temporal ventriloquism compared to the non-congruent sensory pairs (Vatakis and Spence, 2007; Chen and Vroomen, 2013). Concerning the double flash illusion, since the reliability of auditory event is greater than the visual event in the temporal domain, the temporal frequency of auditory beeps perceptually dominated the temporal frequency of visual flashes (Andersen et al., 2004). Moreover, researchers have demonstrated that the double flash illusory percept follows the principles of Bayesian causal inference models by manipulating the relative reliabilities of auditory and visual stimuli (Shams et al., 2005). Similarly, duration estimates of audio-tactile and audio-visual sensory signals are found to be in accordance with the Bayesian causal inference models (Hartcher-O'Brien et al., 2014; Ball et al., 2017). However, previous literature has indicated that the individual multisensory features are either under- or overweighted than expected by Bayesian causal inference in binding due to the inherent limitations in the models (for the detailed review, see Noppeney, 2020). Future studies are required to refine the current Bayesian models to fully account for the multisensory perception.

The Bayesian framework of the multisensory causal inference model became an influential model by systematically explaining the empirical evidence of multisensory perception literature. However, the current multisensory literature indicated that the reported multisensory binding effects are influenced by a combination of more than one factor, and it is not clear how they independently and interactively modulate the multisensory temporal perception. For instance, factors such as spatial and temporal proximity, semantic (or learned) congruency between pairs of cues, and attentional allocation are all known to influence temporal perception (Oever et al., 2016). Future studies are required to orthogonally manipulate these factors within an experimental paradigm in order to understand their independent and interactive roles in the temporal binding phenomenon.

### Bayesian Casual Inference in Motor-Sensory Temporal Binding

The last few decades of research have focused on the temporal processing of multisensory features that are passively received by the study participants. However, in the real-world, multisensory features can also occur because of our interactions with the environment. The broader question is whether the process by which motor-sensory cues generated by voluntary action are bound differs from the passively received sensory cues. Previous literature indicated temporal binding between voluntary motor action and its causal sensory outcome (Haggard et al., 2002; Hughes et al., 2013). For instance, Haggard et al. (2002) indicated the perceived temporal attraction between voluntary action onset (keypress) and its predictable sensory outcome, such as a brief tone (Haggard et al., 2002). In their study, participants were asked to watch a clock face and report when an action was performed and when the sensory outcome was presented in two conditions. In baseline conditions (single event conditions), the study participants reported the onset times of keypress (voluntary action), time of muscle twitch produced by Trans-cranial Magnetic stimulation (TMS condition) on the motor cortex, time of audible sound created by TMS without muscle twitch (TMS sham condition), and time of a tone onset (tone condition) in separate trials. An audible tone appeared in operant conditions after 250 milliseconds of each voluntary keypress condition, TMS, and sham TMS conditions. The task of the subjects was to report the time of both events in operant condition at the end of each trial. Their study results indicated the perceived temporal attraction between action and its outcome (tone) when participants intentionally performed an action rather than TMS-induced involuntary action (Haggard et al., 2002). In other words, action and outcome are bound together by shifting the perceived temporal onsets toward each other when participants intentionally performed an action. Hence, it has been called the “intentional binding” (IB) effect. Further, their study indicated the increased IB effect when the outcome was short delayed after the action and temporally predictable. However, as the delay increased between action and outcome, and the outcome temporally became unpredictable, the IB effect was reduced. This evidence indicates the importance of spatiotemporal factors for causal determination and temporal binding of action and its sensory outcome. The IB effect was attributed to the motor-based predictive mechanisms since IB appeared for voluntary (intentional) and not for involuntary (TMS-induced) actions (Haggard et al., 2002; Hughes et al., 2013). Waszak et al. (2012) proposed a pre-activation account that explains how the sensory action–outcome binds to the action (Waszak et al., 2012). According to the pre-activation account, predicted action–outcomes are pre-activated and increase their baseline neural-activity before the outcome occurs. Since the neural units of predicted outcomes are already activated to some baseline level by the motor-based predictive mechanisms, less strength of the signal is required for reaching the detection threshold. Thus, the action–outcome reaches threshold awareness faster and is perceived temporally closer to the action.

Contrastingly, studies also indicated that IB-like effects appeared even for non-intentional (passive) actions (Buehner, 2015; Borhani et al., 2017; Suzuki et al., 2019), machine-made action and its causal outcome (Buehner, 2012), or observation of other's action and its causal outcome (Poonian et al., 2015). This evidence casts severe doubts on the role of motor-based (forward model) predictive mechanisms on IB and suggests a general predictive mechanism responsible for the temporal binding between action and its sensory outcome (Dogge et al., 2019; Press et al., 2019).

A number of recent studies have begun to investigate IB mechanisms from the perspective of Bayesian cue integration (Moore and Obhi, 2012; Wolpe et al., 2013; Lush et al., 2019). Considering the action and its sensory outcome are causally related, and the temporal judgments of action timing and its outcome are prone to inaccuracies due to the noise, one can model the IB in terms of the Bayesian cue integration framework. For example, Wolpe et al. (2013) manipulated the action outcome's (a brief tone) temporal precision or reliability (inverse of the variance) by adding white noise. They found that the perceived onset time of auditory outcome attracted more to the action when the reliability of the tone was weak (e.g., with added noise) compared to the high-reliability tone (e.g., with no added noise). In another study by Lush et al. (2019), the participants were divided into two groups based on their reliability of time judgments of intentional action (low and high-reliability groups) and measured the perceived temporal attractions between action and its outcome. Their study indicated that the perceived time of action attracted more toward the outcome in the low-reliability group than in the high-reliability group. Legaspi and Toyoizumi (2019) explicitly compared the results of observed IB effects in the studies of Haggard et al. (2002) and Wolpe et al. (2013) with the predictions of the Bayesian cue combination model (Legaspi and Toyoizumi, 2019). Interestingly, their model reliably predicted the intentional binding effects observed in the studies of Haggard et al. (2002) and Wolpe et al. (2013). Concerning the duration aspect of the time dimension, auditory or visual perceived durations are modulated in action contexts (Press et al., 2014; Anobile et al., 2019). However, there is a lack of studies assessing the Bayesian integration of duration estimates in motor-sensory contexts. The abovementioned studies indicated that temporal binding between motor action and its sensory outcome follow general rules of Bayesian cue integration common to the multisensory perceptual phenomenon and not necessarily restricted to the motor-based predictive mechanisms. Future studies are required to evaluate the Bayesian cue integration model to understand how action modulates the temporal binding of multisensory outcomes having differential temporal precisions. This leads to a more naturalistic understanding of the role of action on perception since our actions often produce multiple sensory stimuli.

## Conclusions

This review explored the temporal binding mechanisms in multisensory and motor-sensory contexts. By critically evaluating the recent empirical evidence, this paper suggests that the common computational mechanisms grounded in Bayesian causal inference models are responsible for the temporal binding in multisensory and motor-sensory contexts. Moreover, the extent of temporal binding depends on the strength of prior and the precision of sensory likelihoods. Future studies are required to understand the independent and interactive roles of multiple priors and sensory likelihoods on temporal binding across the multisensory and motor-sensory features.

## Author Contributions

The author confirms being the sole contributor of this work and has approved it for publication.

## Conflict of Interest

The author declares that the research was conducted in the absence of any commercial or financial relationships that could be construed as a potential conflict of interest.
